# Supplementary material for: Drug-disease networks and drug repurposing
Source: PLoS Comput Biol. 2025 Oct 16;21(10):e1013595. doi: 10.1371/journal.pcbi.1013595 (PMC12548869; doi:10.1371/journal.pcbi.1013595)
Supplement: S1 Text — Section 1. Selection of drugs included in the data set. Section 2. Theoretical limits on performance. Section 3. Expectation-maximization algorithm for PLSA. Section 4. Algorithm parameters. Table A. Categories of drugs removed from the data set, along with the number in each category. Note that the total number of drugs removed is less than the sum of the entries in the right-hand column because some drugs belong to more than one category. Table B. Parameters controlling each algorithm. (PDF) [file pcbi.1013595.s001.pdf]

Drug-disease networks and drug repurposing: Supporting Information

Austin Polanco[1] and M. E. J. Newman[1,2*]

[1*]Department of Physics, University of Michigan, Ann Arbor, Michigan, United States of America.
[2]Center for the Study of Complex Systems, University of Michigan, Ann Arbor, Michigan, United States of America.

*Corresponding author(s). E-mail(s): mejn@umich.edu;
Contributing authors: polancoa@umich.edu;

1 Selection of drugs included in the data set

As described in Section 2.1 of the main paper, our data set is assembled from a combination of three pre-existing drug databases, DrugBank, NCATS Inxight, and DailyMed. We start with DrugBank, version 5.1.10, which lists a total of 15 236 distinct medications. Not all of these are of therapeutic interest however. The list also contains things such as cosmetics, foods, allergens, household products, and others, and a crucial step in the preparation of the data is the removal of unwanted entries, which we do in three stages.

First, the DrugBank database assigns each drug to one or more of a set of overlapping status types, including "approved," "investigational," "withdrawn," "illicit," and others. We restrict ourselves to drugs labeled as "approved," a category that denotes

drugs approved for clinical use by the US Food and Drug Administration or one of several comparable agencies in other countries or regions. This removes 10 769 drugs from the data set, more than two-thirds of the initial list.

Second, DrugBank assigns to each drug some number of descriptive labels, including both functional and chemical labels, such as "amino acids," "anti-bacterial agents," or "analgesics." We use these labels to identify entries of minimal therapeutic value, eliminating all those in the categories listed in Table A. This removes an additional 866 entries. There are also some entries in the database that are not listed as belonging to any category. These "unknown" drugs, of which there are 422, we also remove.

These criteria were chosen to focus the network on drugs of established therapeutic value, avoiding non-therapeutic substances. Including the latter could not only lead to the possibility of their diluting more useful predictions, but could also adversely affect the link prediction process itself, making it less accurate. By focusing on entries intended for active well-established disease interventions, the network and resulting predictions should better reflect clinically meaningful drug-disease relationships.

In total we end up removing 12 057 of the starting set of database entries, leaving 3179 core approved drugs in our data set. A final round of hand cleaning of the data reduces this to the 2620 used for the calculations in the paper. During the hand cleaning phase, every interaction in the data set was checked manually to confirm its correctness and a range of other housekeeping operations were performed. This included amalgamating diseases that appeared multiple times in the data set under different names into a single disease. Moreover, other diseases appear in both generic and specific forms and the drugs associated with these were rationalized so that every drug associated with a specific form is also associated with the generic form (e.g., all lung cancer drugs are, by definition, also cancer drugs). In addition, some simple text parsing errors were fixed in the hand cleaning stage, as were (rare) cases of

Category	Entries
Allergenic extracts	23
Bacterial toxins	12
Bee and wasp venom	12
Coloring agents	41
Contrast media	67
Cosmetics	18
Detergents	14
Diagnostic agents	46
Dietary fats	14
Diet, food, and nutrition	92
Food additives	29
Food allergenic extract	195
Food ingredients	29
Fungal allergenic extract	98
Herbs and natural products	31
Household products	16
Metals	72
Mineral supplements	34
Plant allergenic extract	62
Pollen allergenic extract	218
Solvents	20
Standardized chemical allergens	54
Sunscreen agents	27
Sweetening agents	21
Transition elements	38
Venoms	16

Table A Categories of drugs removed from the data set, along with the number in each category. Note that the total number of drugs removed is less than the sum of the entries in the right-hand column because some drugs belong to more than one category.

AI "hallucination," in which the LLM found an indication for a drug that was not supported by the original data.

2 Theoretical limits on performance

Missing data and measurement error can limit the performance of link prediction algorithms in cross-validation tests on real-world data. Even an algorithm that can predict missing edges perfectly will *appear* to fail if it correctly predicts an edge that is missing from the original data set—an action that will not be credited as a correct

prediction in the cross-validation setting. As we show in this appendix, these effects place limits on the maximum value the AUROC score can attain.

Suppose out of all node pairs that are not connected by an edge in our data set, a fraction μ are in fact connected in reality, but those edges are missing from the data set for some reason—because of experimental error or simply because no measurement of them has ever been made. The remaining fraction $1-\mu$ are genuinely not connected by an edge, either in the data set or in reality.

Now we run a cross-validation experiment on the network using some link prediction algorithm. A certain fraction of the observed edges are removed and we attempt to predict them. The link prediction algorithm produces a list of node pairs, drawn from those that are unconnected in the training set, in order from most likely predictions to least likely. Suppose we take the first k entries from the top of this list as our edge predictions. For any value of k, let us define p to be the true positive rate for this k, where we include as "positives" both those edges that were removed for cross-validation and those that were erroneously missing from the data from the outset. And let q to be the corresponding false-positive rate for this value of k.

As we let the value of k vary from zero up to the entirety of the list, the values of p and q describe an ROC curve, but unfortunately this is not a curve we can actually measure, because we do not know the identities of all the edges missing from the data set. We know some of them—the ones that we ourselves removed for cross-validation—but not the ones that were missing from the outset. Still, if we did somehow know all the missing edges then we could calculate the area A_0 under the curve from

$$A_0 = \int_0^1 p \, \mathrm{d}q. \tag{S1}$$

Since we do not know the identity of the edges that are missing from the outset, any link predictions that identify these edges will be wrongly labeled in our cross-validation tests as false positives, when really they are true positives. If we make the assumption that our algorithm is equally good at predicting both the edges removed for cross-validation and edges missing from the outset, then this implies that the estimated true positive rate, as calculated over the edges removed for cross-validation, will remain unchanged at p, but the false positive rate will be exaggerated and will now take on the larger value

$$q' = \mu p + (1-\mu)q. \tag{S2}$$

Here the term μp represents the genuine missing edges that are predicted to be present with probability p but then wrongly labeled as false positives, while the $(1-\mu)q$ represents actual false positives—non-edges that are falsely predicted with probability q.

It is worth pausing for a moment over our assumption that the algorithm is equally good at predicting both types of edges. While this is a reasonable baseline assumption, it could be violated under some circumstances. For instance, it could be that the edges missing from the data set are missing precisely because they are harder to predict: perhaps all the easy-to-find edges have already been added to the data set and those left in the "missing" set are the ones for which link prediction works poorly. Here we assume this not to be the case, so that the average probability of all true positive predictions takes the same value p as it does for the edges removed for cross-validation.

With this assumption, the AUROC value that we calculate in our cross-validation experiment is

$$A = \int_0^1 p\,\mathrm{d}q' = \mu \int_0^1 p\,\mathrm{d}p + (1-\mu) \int_0^1 p\,\mathrm{d}q = \tfrac{1}{2}\mu + (1-\mu)A_0, \tag{S3}$$

where we have used Eq. (S1) in the last equality. The largest possible value of A_0, if we had a perfect prediction algorithm, would be 1. Hence an upper bound on the actual measured area under the curve is

$$A \leq 1 - \tfrac{1}{2}\mu. \tag{S4}$$

Thus, for example, if 10% of true edges are missing from the data set, it will be impossible for any algorithm to achieve an AUROC of greater than $1 - \frac{1}{2} \times 0.1 = 0.95$. Alternatively, we can reverse the argument and say that if we observe an AUROC of A then the fraction μ of edges that could be missing from the data set satisfies

$$\mu \leq 2(1 - A). \tag{S5}$$

This inequality gives us an upper bound on what we can extract from a given network. It tells us that the fraction μ of unobserved edges remaining to be found in the network is always less than $2(1 - A)$, where A is the value of the AUROC statistic measured for any link prediction algorithm. Note that the inequality does not depend on the fraction of edges removed in cross-validation, so one is at liberty to try any fraction one wants, as well as any algorithm one wants, in order to make the AUROC as large as possible and hence obtain the tightest bound.

The inequality satisfies some basic sanity tests. The smallest value of A is $\frac{1}{2}$, which gives $\mu < 1$, implying that potentially all edges are true predictions in this case. This is trivially correct: if all edges were true predictions then even a perfect algorithm would be unable to distinguish between genuinely missing edges and edges removed in cross-validation, so by definition $A = \frac{1}{2}$. Conversely, the largest possible value of A is 1, implying perfect success at predicting the removed edges. This would give us $\mu = 0$, meaning that none of our predictions correspond to genuinely missing edges, which

again is correct: if your algorithm is 100% correct at picking out the edges removed in cross-validation, then there can be no other correct predictions diluting the results.

The presence of missing edges in the data also affects the precision, although in a relatively simple way: the number of true positive predictions is reduced by a factor of $1-\mu$ and hence the precision is reduced by the same factor, as are measures proportional to precision such as AUPR and top-k precision. From (S4) and the fact that μ is positive we have $2A-1 \le 1-\mu \le 1$, which in practice places relatively tight bounds on $1-\mu$ and hence tells us that precision values are not going to be greatly affected by missing edges.

Returning to AUROC values, suppose now that, in addition to the missing edges in the original data (false negatives) there are also false positives: a fraction ν of the observed edges are wrong. Again we run our cross-validation test, removing some of the edges in the data, including some of these false edges, then attempt to predict the removed edges. Predictions of the false edges will occur with probability q, not probability p, and hence the true-positive rate estimated in the experiment will be modified to a new value

$$p' = (1-\nu)p + \nu q, \tag{S6}$$

while the false-positive rate still has the same value as before, Eq. (S2), so our AUROC score becomes

$$\begin{aligned} A &= \int_0^1 p' \, \mathrm{d}q' \\ &= \mu(1-\nu)\int_0^1 p \, \mathrm{d}p + (1-\mu)(1-\nu)\int_0^1 p \, \mathrm{d}q + \mu\nu \int_0^1 q \, \mathrm{d}p + (1-\mu)\nu \int_0^1 q \, \mathrm{d}q. \end{aligned} \tag{S7}$$

Integrating by parts, we have

$$\int_0^1 q \,\mathrm{d}p = \left[pq\right]_{p=0}^{1} - \int_0^1 p \,\mathrm{d}q = 1 - A_0, \tag{S8}$$

and hence (S7) becomes

$$\begin{aligned} A &= \tfrac{1}{2}\mu(1-\nu) + (1-\mu)(1-\nu)A_0 + \mu\nu(1-A_0) + \tfrac{1}{2}(1-\mu)\nu \\ &= \tfrac{1}{2}(\mu+\nu) + [1-(\mu+\nu)]A_0. \end{aligned} \tag{S9}$$

We don't know the sign of the coefficient $1-(\mu+\nu)$ in this expression—it could be either positive or negative—but if we assume the fractions of false positives ν and false negatives μ to be small enough that $\mu+\nu<1$, then the coefficient is positive and setting $A_0 = 1$ again gives us an upper bound $A \le 1 - \frac{1}{2}(\mu+\nu)$ and hence

$$\mu + \nu \le 2(1-A), \tag{S10}$$

which is a generalization of Eq. (S5). This expression places a bound on the sum of the fractions of false positives and false negatives in the data. Since both fractions are positive, it also implies that $\mu \le 2(1-A)$, so our result from before holds even in the presence of false positives in the data. Moreover we also have $\nu \le 2(1-A)$, so we have an upper bound on the fraction of false positives that could be present.

3 Expectation-maximization algorithm for PLSA

The expectation-maximization (EM) algorithm we use for fitting the PLSA model is a bipartite version of the one proposed in [1]. We assume a bipartite network with m, n nodes of types 1 and 2 respectively and a number of edges between node u of type 1 and node v of type 2 that follows a Poisson distribution with mean $\mathbf{r}_u \cdot \mathbf{s}_v =$

$\sum_{z=1}^{K} r_{uz}s_{vz}$, where $\mathbf{r}_u$ and $\mathbf{s}_v$ are K-dimensional vectors with non-negative elements. The assumption of a Poisson distribution may seem surprising, since our drug-disease network only ever has either zero or one edges between any pair of nodes, but in a sparse setting such as ours there is little difference between a Poisson random variable and a zero-one Bernoulli variable, and the Poisson choice is a practical one that makes the calculations simpler.

There is an ambiguous multiplicative factor between r_{uz} and s_{vz} that prevents them from being fully identifiable: for any set of non-negative numbers x_z with $z = 1 \ldots K$, if we multiply r_{uz} by x_z for all u and divide s_{vz} by the same quantity for all v, then the expected number of edges between every pair of nodes remains the same, regardless of the values of the x_z. Here we remove this ambiguity by enforcing the condition

$$\sum_{u} r_{uz} = \sum_{v} s_{vz} \tag{S11}$$

for all z.

Our purpose with this model is to fit it to our observed bipartite network and hence extract the values of the $\mathbf{r}_u$ and $\mathbf{s}_v$, which we can use to estimate the probability of an edge between any two nodes. We perform the fit by the method of maximum-likelihood. Let $\mathbf{B}$ be the $m \times n$ incidence matrix of the network with elements $B_{uv} = 1$ if there is an edge between nodes u and v and 0 otherwise. Then the likelihood of the network is a product of Poisson distributions:

$$P(\mathbf{B}|\mathbf{r}, \mathbf{s}) = \prod_{u=1}^{m} \prod_{v=1}^{n} \frac{(\mathbf{r}_u \cdot \mathbf{s}_v)^{B_{uv}}}{B_{uv}!} e^{-\mathbf{r}_u \cdot \mathbf{s}_v}, \tag{S12}$$

and the log-likelihood is

$$\log P(\mathbf{B}|\mathbf{r}, \mathbf{s}) = \sum_{uv} \left[B_{uv} \log \sum_{z=1}^{K} r_{uz}s_{vz} - \sum_{z=1}^{K} r_{uz}s_{vz} \right], \tag{S13}$$

where we have written out the dot products in full and made use of the fact that $B_{uv} = 0$ or 1 so that $B_{uv}! = 1$ for all u, v. Now we employ Jensen's inequality in the form $\log \sum_i x_i \geq \sum_i q_i \log(x_i/q_i)$, where the x_i are any set of positive reals and the q_i are any set of positive reals that sum to 1. Applying this inequality to the first term in (S13) we get

$$\log P(\mathbf{B}|\mathbf{r},\mathbf{s}) \geq \sum_{uvz} \biggl[B_{uv} q_{uv}(z) \log \frac{r_{uz} s_{vz}}{q_{uv(z)}} - r_{uz} s_{vz} \biggr], \tag{S14}$$

where $q_{uv}(z)$ is any set of positive quantities such that $\sum_z q_{uv}(z) = 1$.

The exact equality is achieved when

$$q_{uv}(z) = \frac{r_{uz} s_{vz}}{\sum_z r_{uz} s_{vz}}, \tag{S15}$$

meaning also that this choice maximizes the right-hand side of (S14) with respect to $q_{uv}(z)$. Thus by maximizing with respect to $q_{uv}(z)$ and then maximizing the resulting expression with respect to $\mathbf{r}_u$ and $\mathbf{s}_v$ for all u, v we obtain the maximum-likelihood solution we seek. To put that another way, a double maximization of the right-hand side of (S14) with respect both to $q_{uv}(z)$ and to $\mathbf{r}_u, \mathbf{s}_v$ will achieve our goals. In the EM algorithm we perform this double maximization by simply maximizing repeatedly and alternately with respect to $q_{uv}(z)$ using Eq. (S15) and with respect to $\mathbf{r}_u, \mathbf{s}_v$ by differentiation.

Differentiating the right-hand side of (S14) with respect to r_{uz} and s_{vz} for fixed $q_{uv}(z)$ gives us the two equations

$$r_{uz} = \frac{\sum_v B_{uv} q_{uv}(z)}{\sum_v s_{vz}}, \qquad s_{vz} = \frac{\sum_u B_{uv} q_{uv}(z)}{\sum_u r_{uz}}. \tag{S16}$$

Summing the first of these over u and using Eq. (S11) gives

$$\sum_u r_{uz} \sum_v s_{vz} = \biggl(\sum_u r_{uz}\biggr)^2 = \biggl(\sum_v s_{vz}\biggr)^2 = \sum_{uv} B_{uv} q_{uv}(z), \tag{S17}$$

and hence

$$\sum_u r_{uz} = \sum_v s_{vz} = \sqrt{\textstyle\sum_{uv} B_{uv} q_{uv}(z)}. \tag{S18}$$

Then (S16) becomes

$$r_{uz} = \frac{\sum_v B_{uv} q_{uv}(z)}{\sqrt{\sum_{uv} B_{uv} q_{uv}(z)}}, \qquad s_{vz} = \frac{\sum_u B_{uv} q_{uv}(z)}{\sqrt{\sum_{uv} B_{uv} q_{uv}(z)}}. \tag{S19}$$

Note that because $q_{uv}(z)$ always appears in the combination $B_{uv} q_{uv}(z)$ it is only necessary to calculate it for node pairs u, v that are joined by an edge in the network. Values for all other u, v contribute zero to the sums.

We now have a complete algorithm for estimating r_{uz} and s_{vz}. Starting from an initial guess at their values (such as random numbers), we compute $q_{uv}(z)$ from Eq. (S15) for all z and all u, v connected by an edge, then we calculate updated values of r_{uz} and s_{vz} from Eq. (S19), and repeat the whole process until convergence is achieved. If there are M edges in total in the network then the running time is $\mathrm{O}(KM)$ per round of the algorithm, and total running time is this amount times the number of rounds.

Finally, to perform link prediction, we calculate a prediction score within the fitted model for each possible node pair u, v as the expected number of edges $\sum_z r_{uz} s_{vz}$ between those nodes, and generate edge predictions in order from highest score to lowest.

Algorithm	Parameters
SVD	embedding dimension = 60
PLSA	embedding dimension = 90
NNMF	embedding dimension = 80
Node2vec	embedding_dim = 256, walk_length = 20, context_size = 20, walks_per_node = 40, batch_size = 128, num_epochs = 200, lr = 0.01, p = 1.0, q = 1.0
GEBE^p	embedding size = 96, heat constant = 1
BPR	no_components = 100, num_epochs = 650, learning_rate = 0.01
ICTC	model1 = LGAE, model2 = GAE, learning_rate = 0.01, num_epoch = 200, hidden1_dim = 32, hidden2_dim = 16, numexp = 10

Table B Parameters controlling each algorithm.

4 Algorithm parameters

The behavior of some of the algorithms we study is controlled by various user-determined parameters, listed in Table B along with the values used in our calculations. These values were in general chosen by performing a coarse search over the parameter space to optimize prediction performance according to the AUPR measure. Some parameters were found to have a negligible impact on performance and these were left at their default or canonical values. Across all algorithms, the most parameter whose variation had the greatest effect was the dimension of the learned embedding, which has a substantial effect on AUPR values.

References

[1] Ball, B., Karrer, B., Newman, M.E.J.: An efficient and principled method for detecting communities in networks. Phys. Rev. E **84**, 036103 (2011)
